# Autism Spectrum Disorders and Perinatal Complications—Is Oxidative Stress the Connection?

**DOI:** 10.3389/fpsyt.2019.00675

**Published:** 2019-09-25

**Authors:** Vanja Mandic-Maravic, Marija Mitkovic-Voncina, Marija Pljesa-Ercegovac, Ana Savic-Radojevic, Miroslav Djordjevic, Tatjana Pekmezovic, Roberto Grujicic, Marko Ercegovac, Tatjana Simic, Dusica Lecic-Tosevski, Milica Pejovic-Milovancevic

**Affiliations:** ^1^Institute of Mental Health, Belgrade, Serbia; ^2^Faculty of Medicine, University of Belgrade, Belgrade, Serbia; ^3^Institute of Medical and Clinical Biochemistry, Belgrade, Serbia; ^4^University Children’s Hospital, Belgrade, Serbia; ^5^Institute of Epidemiology, Belgrade, Serbia; ^6^Serbian Academy of Sciences and Arts, Belgrade, Serbia

**Keywords:** autism, perinatal complications, oxidative stress, prematurity, glutathione transferase

## Abstract

**Background:** Autism spectrum disorders (ASD) are complex psychiatric disorders, with gene environment interaction being in the basis of their etiology. The association of perinatal complications and ASD is well established. Recent findings suggested that oxidative stress and polymorphism in genes encoding antioxidant enzymes might be involved in the development of ASD. Glutathione transferases (GSTs) have an important role in the antioxidant defense system. We aimed to establish whether the predictive effects of prenatal and perinatal complications (as possible oxidative stress inducers) on ASD risk are dependent on GST polymorphisms.

**Methods:** The study included 113 ASD cases and 114 age- and sex group-matched healthy controls. All participants were genotyped for GSTA1, GSTM1, GSTT1, and GSTP1 polymorphisms. The questionnaire regarding prenatal and perinatal risk factors and complications was administered for all the subjects in the study.

**Results:** The evaluated perinatal complications as a group significantly increased the risk of ASD [odds ratio (OR) = 9.415; p = 0.000], as well as individual perinatal complications, such as prematurity (OR = 11.42; p = 0.001), neonatal jaundice (OR = 8.774; p = 0.000), respiratory distress syndrome (OR = 4.835; p = 0.047), and the use of any medication during pregnancy (OR = 2.413; p = 0.03). In logistic regression model, adding GST genotypes did not modify the significant effects found for prematurity and neonatal jaundice as risk factors in ASD. However, there was a significant interaction of GST genotype with medication use during pregnancy and the use of tocolytics during pregnancy, which was predictive of ASD risk only in carriers of *GSTM1-null*, as opposed to carriers of *GSTM1-active* genotype.

**Conclusion:** Specific perinatal complications may be significant risk factors for ASD. *GSTM1* genotype may serve as a moderator of the effect of some prenatal factors on the risk of ASD such as using medication during pregnancy. It may be speculated that different oxidative stress-related genetic and environmental factors could lead to development of ASD. Apart from etiological mechanisms, possible therapeutic implications in ASD are also discussed.

## Introduction

The increasing prevalence of autism spectrum disorder (ASD) has led to an increase in interest for environmental factors and their potential influence (1). By definition, environmental risk factors are those non-genetic factors that lead to development of a disorder in individuals with a genetic susceptibility (2). Recognizing the impact of environmental factors, as well as gene–environment interactions in persons at risk, may be of great importance for prevention and treatment of ASD (3, 4)

Multiple studies explored the effect of prenatal and perinatal factors on the risk of ASD. They explored various factors, using different criteria, and getting different results (5).

Maternal age was found to be significant in several large studies, (6, 7), while the most recent study showed a different finding—the risk for ASD might increase if the mother is younger (8). It is argued that maternal age might have a direct effect on ASD risk—possibly by epigenetic changes (9), and also might increase the risk for perinatal complications in general (10). The results for paternal age and the risk of ASD were more consistent—a larger number of studies showed it might be significant risk factor (6, 7, 11–13), although there are studies with different results (5).

The effect of medication during pregnancy is well established (8, 13, 14). The studies exploring the use of medication during pregnancy and the risk of ASD were mostly oriented towards mood stabilizers and antidepressants (15, 16). There are only few studies exploring the effect of other medication during pregnancy. A meta-analysis done in 2009 also showed that the use of medication during pregnancy increased risk of ASD, 1.46 times. Also, the study by Dodds et al. confirmed that the risk for ASD increases 2.66 times with prescribed medication (in this study mostly lithium, antihypertensives, antidepressants, and anticoagulants) (17).

Prematurity was also identified as a significant risk factor for ASD (5, 12, 13, 18). Surprisingly, a recent large study did not prove prematurity to be a significant risk factor for this group of disorders (8). Asphyxia at birth as well as respiratory distress syndrome (RDS) have also been established as ASD risk factors (5, 12, 17, 19, 20). Several studies confirmed low birth weight (LBW) to be a significant ASD risk factor as well (21, 22), but other results were conflicting (5). Conflicting results were also found for intracranial hemorrhage (12, 17, 19). Significant findings were shown for neonatal jaundice and risk for ASD as well (5, 23–25), while several studies did not show this significance (8, 11).

Oxidative stress is proposed to be important in the etiology of ASD, and it might be the underlying mechanism by which prenatal and perinatal complications possibly contribute to ASD development (3). The transition from fetal to neonatal phase is a great stress for the newborn due to a significant increase in the production of free radicals. Mature and healthy newborns overcome these changes in oxygen concentration, but the problem may occur when intrauterine development is derranged in some way (26). Increased levels of oxidative stress were found in newborns with RDS (26, 27). In prematurity, one of the most common types of brain injury is diffuse white matter injury (DWMI), and studies have shown that it is oxidative stress related (28, 29). Recent studies have shown oxidative distress in children with hyperbilirubinaemia, manifested as decreased levels of paraoxonase and increased levels of malondialdehyde (30, 31). Also, it was found that markers of oxidative stress in newborns with hyperbilirubinemia [decreased glutathione (GSH)] tend to normalize after phototherapy and lowering in bilirubin concentration (32).

The effect of parental age on ASD also might be explained by the effects of oxidative stress (33). The spermatosoids of older men have a higher level of DNA damage, due to higher sensitivity to oxidative stress—offspring of older men may have more DNA fragmentation that lead to neuropsychiatric disorders (33). Also, older women might have lower capacity for homocystein cycle, leading to a decreased antioxidant defense in the embrion. This series of events also migh lead to neuropsychiatric disorders in the offspring (33).

It has been suggested that certain genetic polymorphisms might make children more vulnerable to perinatal complications (29). Studies that explored the oxidative stress as the basis of the gene–environment interaction in ASD also pointed out to the possible role of glutathione transferases (GSTs) in ASD development, especially regarding their important role in the antioxidant defense system (33–36). Several studies have proposed the significant association between GST polymorphisms and ASD risk, either independent (37) or in interaction with environmental factors, such as exposure to lead, mercury, and aluminum (35, 36). Our recent results have shown that *GSTM1 active* genotype decreases the risk of ASD, while *GSTA1 CC* genotype increases susceptibility to ASD. The combination of *GSTM1 active* and *GSTT1 active* as well as combination of *GSTT1 active* and *GSTP1 llelle* genotypes decrease the risk for ASD, while a higher risk of ASD was observed if combination of *GSTM1 active* and *GSTP1 llelle* was present (38).

In this study, we explored the frequency of specific prenatal and perinatal complications in patients with ASD and healthy controls, determining their effect on the risk for ASD. We further aimed to establish whether the predictive effects of prenatal and perinatal complications are affected by the most common GST polymorphisms (GSTA1, GSTM1, GSTP1, and GSTT1), in order to explore the possible complex multifactorial etiological pathway for developing ASD.

## Materials and Methods

### Study Population

This was a case–control study, involving 113 ASD patients (92 males, 21 females, 9.36 ± 5.88 years old) and 114 age- and sex group-matched controls. The inclusion criterion for the case group was any of the ASD diagnosis. The diagnosis was made using the ICD-10 criteria (39), confirmed by a child psychiatrist with experience in diagnosing and treatment of ASD. The evaluation was performed in a clinical interview with a parent and examination of a child. Besides clinical assessment, the diagnosis was confirmed by the Autism Diagnostic Interview—Revised (ADI-R) (40), conducted by a trained child psychiatrist.

The control group was recruited from the Urology and Orthopedic Department of University Children’s Hospital, Belgrade, Serbia. Control subjects were diagnosed with unintentional injuries (fractures) and urogenital tract disorders (phimosis, chryptorchismus, penal curvature), and were recruited consecutively, at the same time as the cases. The exclusion criteria for the controls were presence of a neurological or psychiatric disorders as well as any kind of developmental delays in personal or family history. The difference in age and sex distribution within the group level was not statistically significant.

### Instruments

*Autism Diagnostic Interview—Revised (ADI-R)* (40). ADI-R is a standardized semi-structured parent/caregiver interview, used for the assessment of signs of ASD. The description of each item, given by the parent/caregiver, is made for childhood (ever) and current behavior. Specific items regarding social reciprocity, communication, and restricted, repetitive, and stereotyped behavior (RRSB) are used to create the scores for these three domains (ADI-R A, ADI-R B, and ADI-R C score, respectively). Higher scores mean greater impairment—more severe symptoms. In this study, the interview was performed by certified child psychiatrists.

*Sociodemographic and exposure questionnaire* was created for the current study and was administered to parents of cases and controls. Aside from the basic sociodemographic information, the questionnaire examined different prenatal exposures, as well as perinatal complications in participants of the study (parental age, parity, infections, smoking, alcohol intake during pregnancy, prematurity, neonatal jaundice, RDS, intracranial hemorrhage, etc.). It comprised questions regarding both the presence and quantity of specific exposure/complication. The questionnaire is shown in the **Supplemental Material**.

### DNA Isolation

Total DNA was isolated from 200 μl of the whole peripheral blood using QIAamp DNA Blood Mini Kit (Qiagen, Chatsworth CA, USA) and QIAamp Mini spin columns with a small chance of sample-to-sample cross-contamination. In the first step, optimized detergent buffers and enzyme Proteinase K (600 mAU/ml, 40 mAU/mg protein) were used to lyse samples and stabilize DNA. In the second step, DNA was adsorbed onto the QIAamp silica membrane during a brief centrifugation. The lysate buffering conditions are adjusted to allow optimal binding of the DNA to the QIAamp silica membrane. In the following two steps, the DNA bound to the QIAamp membrane was washed without affecting DNA binding. Purified DNA was eluted from the QIAamp Mini spin column in a concentrated form in AE Buffer. Isolated DNA, free of protein, nucleases, and other contaminants or inhibitors, was stored at −20°C for later use. DNA concentration and purity were determined spectrophotometrically at 230, 260, 280, and 320 nm using GeneQuant pro (Biochrom, Cambridge, England).

### GST Genotyping

Genotyping was performed blinded to the case–control status, and blinded quality control samples were inserted to validate genotyping identification procedures. Concordance for blinded samples was 100%. All assays performed contained positive and negative controls.

**Figure 1:**
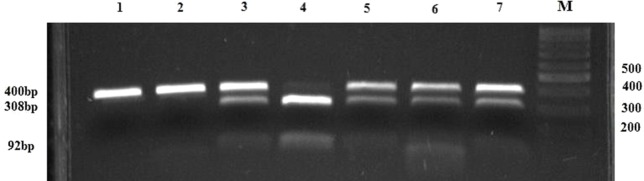
PCR-RFLP restriction products of the *GSTA1* gene. Lanes 1 and 2 represent PCR products of GSTA1*CC genotype (400 bp bands); lanes 3, 5, 6, and 7 represent PCR-RFLP restriction products of GSTA1*CT genotype (400 bp, 308 bp, 92 bp bands); Lane 4 comprises RFLP-PCR restriction products of GSTA1*TT genotype (308 bp, 92 bp bands); M, DNA marker; N, negative control without a DNA content.

The analysis of the SNP GSTA1 -69C > T (rs3957357) was performed using polymerase chain reaction (PCR)–restriction fragment length polymorphism (RFLP) according to the method by Ping et al. (41). A 400 bp fragment was amplified in a reaction mixture containing primers, MasterMix, and water (Thermo Fisher Scientific, Waltham, Massachusetts, USA) and subjected to the PCR protocol indicated in the **Table 1**. For RFLP analysis, 5 μl of PCR product was digested overnight at 37°C with 2 U of restriction enzyme EarI and 1xTango Buffer (Thermo Fisher Scientific, Waltham, Massachusetts, USA) in total volume of 15 μl. DNAse free water was used as the negative control. Digested products (GSTA1*CC: 400 bp, GSTA1*CT: 400 bp + 308 bp + 92 bp and GSTA1*TT: 308 bp + 92 bp) were separated on 3% agarose gel (125 V constant, 0.27 A, 50 W) and stained with SYBR^®^ Safe DNA Gel Stain (Invitrogen Corporation, Carlsbad, CA, USA) and visualized on GL200 Camera (Gel Logic Imaging System, Kodak) or on Chemidoc (Biorad, Hercules, CA, USA) (**Figure 1**).

**Table 1:** The primer sequences, PCR conditions, and restriction enzymes.

Polymorphism	Primer sequences	PCR protocol
GSTA1*C-69T		Denature: 94˚C for 3 min
	F, 5′-GCATCAGCTTGCCCTTCA -3′, R, 5′-AAACGCTGTCACCGTCCTG -3′	Followed by 94˚C for 30 s Annealing: 56˚C for 30 s Extension: 72˚C for 30 s #cycles: 30 Final extension: 72˚C for 10 min Restriction enzyme: Eam1104I incubation at 37˚C overnight
GSTP1*Ile105Val	F, 5′-ACCCCAGGGCTCTATGGGAA-3′, R, 5′-TGAGGGCACAAGAAGCCCCT-3′	Denature: 95˚C for 10 min Followed by 94˚C for 30 s Annealing: 59˚C for 30 s Extension: 72˚C for 30 s #cycles: 29 Final extension: 72˚C for 10 min Restriction enzyme: Alw26I incubation at 37˚C overnight
*GSTM1*	F, 5′-GAACTCCCTGAAAAGCTAAAGC-3′, R, 5′-GTTGGGCTCAAATATACGGTGG-3′	Multiplex PCR: Denature: 94˚C for 3 min
*GSTT1*	F, 5′-TTCCTTACTGGTCCTCACATCTC-3′, R, 5′-TCACGGGATCATGGCCAGCA-3′	Followed by 94˚C for 30 s Annealing: 59˚C for 30 s Extension: 72˚C for 45 s #cycles: 30 Final extension: 72˚C for 4 min
*CYP1A1*	F, 5’-GAACTGCCACTT CAGCTGTCT-3’ R, 5’-CAGCTGCATTTG GAAGTGCTC-3’	

**Figure 2:**
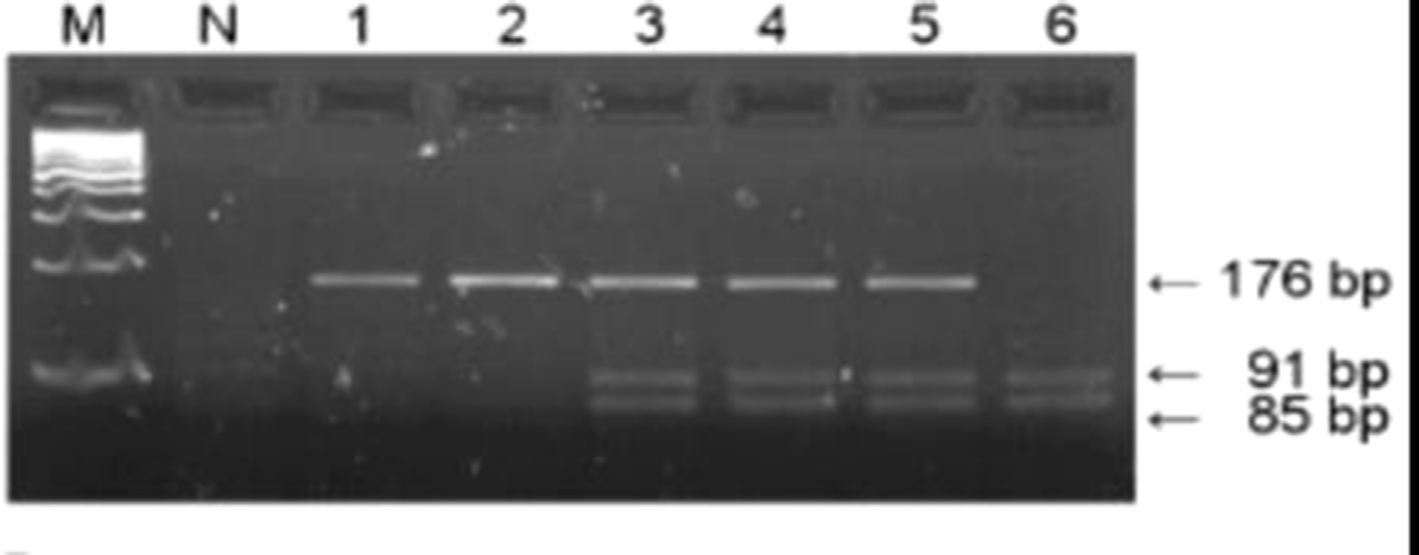
PCR–RFLP restriction products of the *GSTP1* gene. Lanes 1 and 2 represent products of wild-type (Ile/Ile) genotype, lanes 3, 4, and 5 represent heterozygous (Ile/Val) while lane 6 indicates homozygous (Val/Val) genotype; M, DNA Q2 marker; N, negative control.

GSTP1 Ile105Val polymorphism was analyzed using the PCR–RFLP method by Harries et al. (42). Briefly, amplification was conducted using primers presented in **Table 1**. The amplification was performed by denaturing at 95°C for 10 min, followed by 29 cycles at 94°C for 30 s, annealing at 59°C for 30 s and 72°C for 30 s. The final extension was done at 72°C for 10 min. The amplification 176 bp products were digested by 10 U of restriction endonuclease Alw261 (Thermo Fisher Scientific, Waltham, Massachusetts, USA) at 37°C overnight and eletrophoresed on 3% agarose gel. The presence of restriction site resulting in two fragments (91 and 85 bp) indicated variant allele (Val/Val), while presence of only 176 bp fragment indicated Ile allele (Ile/Ile). In case of heterozygous genotype (Ile/Val), all three fragments were present (**Figure 2**).

**Figure 3:**
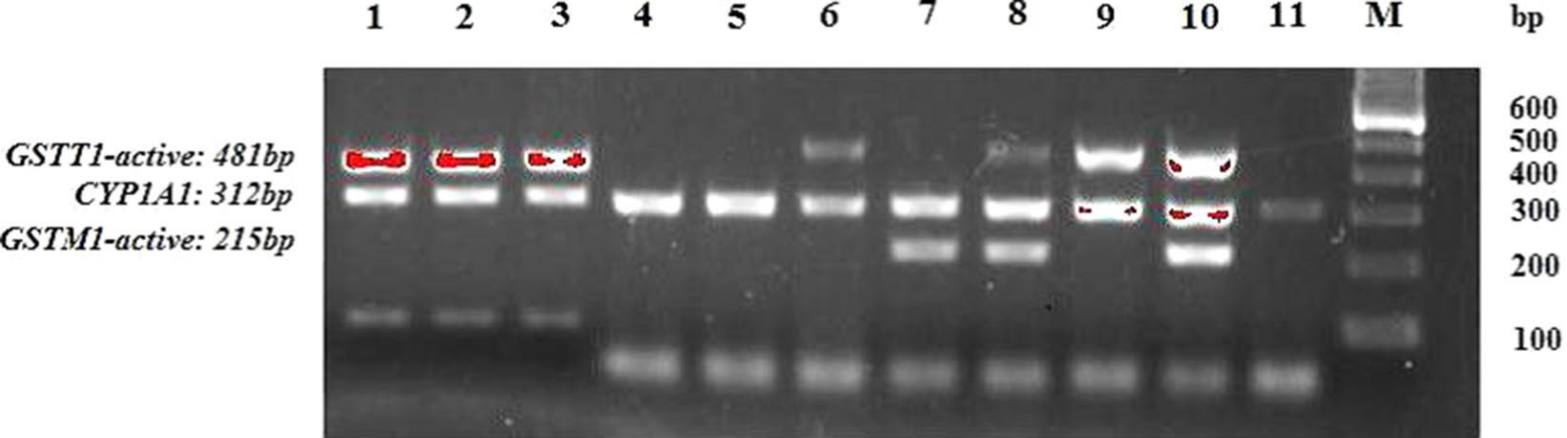
PCR products of the *GSTM1* and *GSTT1* genes. Lanes 8 and 10 comprise PCR products of combined *GSTT1-active/GSTM1-active* genotype (481 bp and 215 bp bands, respectively); lanes 1, 2, 3, 6, and 9 comprise PCR products of *GSTT1-active/GSTM1-null* genotype (481 bp bands); Lane 7 represents PCR products of *GSTT1-null/GSTM1-active* genotype (215 bp bands); Lines 4, 5, and 11 indicate *GSTT1-null/GSTM1-null* genotype; 312 bp band represents the *CYP1A1* housekeeping gene, used as internal control for amplifiable DNA; M, DNA marker.

The DNA sequences of GSTM1 and GSTT1 were analyzed by multiplex PCR in Mastercycler gradient thermal cycler (Eppendorf, Hamburg, Germany) according to the method by Abdel-Rahman et al. (43). The multiplex PCR technique used to detect homozygous deletions of GSTM1 and GSTT1 included primers for GSTM1, GSTT1, and CYP1A1 housekeeping gene, used as an internal control for amplifiable DNA (**Table 1**). Isolated DNA (∼50 ng) was amplified in a total volume of 25 μl reaction mixture containing 7.5 pmol of each primer, 12.5 μl of MasterMix (0.05 U/μl Taq DNK polymerase, 4 mmol MgCl_2_, 0.4 mmol of dNTP) and water (Thermo Fisher Scientific, Waltham, Massachusetts, USA). Amplified PCR products (GSTM1: 215 bp, GSTT1: 481 bp, CYP1A1: 312 bp) were electrophoresed (125 V constant, 0.27 A, 50 W) on 2% agarose gel, stained with SYBR^®^ Safe DNA Gel Stain (Invitrogen Corporation, Carlsbad, CA, USA), and visualized on GL200 Camera (Gel Logic Imaging System, Kodak) or on Chemidoc (Biorad, Hercules, CA, USA) (**Figure 3**). Since the assay does not distinguish heterozygous or homozygous wild-type genotypes and therefore detects the presence (at least one allele present, homozygote or heterozygote) or the absence (complete deletion of both alleles, homozygote) of the genotype, the active genotype was detected according to presence of the particular band (GSTM1-active: 215 bp, GSTT1-active: 481 bp) and the absence of these bands was indicative of the null genotypes.

### Statistical Analysis

Beside descriptive statistics, the study included the tests of statistical difference of control variables between the case and the control group (χ^2^ or t test depending on the variable type). The χ2 test was also used for the assessment of possible genotype departure from Hardy–Weinberg equilibrium. Series of univariate logistic regressions were conducted for all explored perinatal and prenatal factors in order to produce univariate odds ratios (OR). These were followed by a two-step multivariate logistic regression model of ASD risk. The first step included prenatal and perinatal predictors found in univariate analyses, which were the most present in the sample. In this step, we were also controlling for familial factors (parental age) due to its possible effect on perinatal complications in general and its link to oxidative stress (10, 33). In the second step, these predictor effects were adjusted for the GST genotypes. Since one variable effect (use of medication during pregnancy) lost its significance after the second step, a moderation effect of GST genotypes on this variable was analyzed using moderation analyses [based on ordinary least square regression within path analysis method, using bootstrapping confidence intervals (CIs) - macro PROCESS] (44). The moderation analysis was conducted for the use of tocolytics during pregnancy as well. As effect size indicators, we used OR (with the 95% CI), as well as Cox & Snell and Nagelkerke R^2^. The probability level of ≤0.05 was considered statistically significant.

### Ethical Standards

The study was approved by the Ethics Committee of the Institute of Mental Health, University Children’s Hospital and Faculty of Medicine, University of Belgrade, Serbia, and has been performed in accordance with the principles of good research practice. Prior to participation in the study, parents/caretakers signed the informed consent.

## Results

Basic sociodemographic characteristics of ASD cases and controls are shown in **Table 2**. There were no differences in age and sex between the case and the control group (p = 0.120 and p = 0.731, respectively). There were no differences in maternal age (p = 0.465) or paternal age (p = 0.159) as well. Finally, there were no significant differences in maternal and paternal education between ASD cases and controls (p = 0.100 and p = 0.793, respectively).

**Table 2:** Sociodemographic factors in ASD cases and controls.

Variable	Cases (n = 113)	Controls (n = 114)	t	X^2^	P
**Child’s age (years),** **X ± SD**	9.36 ± 5.88	10.62 ± 6.33	−1.562	/	0.120
**Child’s sex, n (%)**					
Male Female	92 (81) 21 (19)	95 (83) 19 (17)	/	0.144	0.731
**Maternal age at birth**	28.45 ± 4.79	27.93 ± 5.42	0.731	/	0.465
**Paternal age at child’s birth**	32.93 ± 6.32	31.69 ± 6.12	1.415	/	0.159
**Mother’s educations**					
Elementary school High school More than high school	10 (9.9) 43 (42.6) 48 (47.5)	5 (4.9) 58 (56.3) 40 (38.8)	/	4.602	0.100
**Father’s education**					
Elementary school High school More than high school	8 (8.1) 57 (57.6) 34 (34.3)	8 (7.8) 64 (62.1) 31 (30.1)	/	0.464	0.793

Perinatal complications of ASD cases and respective controls are shown in **Table 3**. Comparing to control group, the group of ASD cases had higher frequency of medication use during pregnancy (p = 0.030, OR = 2.413; CI: 1.35–4.32), particularly the tocolytics (p = 0.029; OR = 2.467; CI: 1.098–5.546). Also, significant differences were shown for perinatal complications. Having any perinatal complication raised the risk of ASD 9.415 times (p = 0.000; OR = 9.415; CI: 4.870–18.203). Prematurity (p = 0.001; OR = 11.42; CI: 2.586–50.455), neonatal jaundice (p = 0.000, OR = 8.774; CI: 4.11–18.725), and RDS (p = 0.047, OR = 4.835; CI: 1.018–22.957) were significantly more present in the case group. There were no significant differences in the frequency of LBW (p = 0.205), perinatal asphyxia (p = 0.209), or intracranial hemorrhage (p = 0.169) between cases and controls. No differences were observed in the parental age either.

**Table 3:** Prenatal and perinatal complications in the case and the control group—descriptives and univariate analyses.

Variable	Cases	Controls	X^2^/t	Sig.	Univariate logistic regression OR	Sig.
Use of medication during pregnancy (any)	Yes 48 (47.1%) No 54 (52.9%)	Yes 28 (36.8%) No 76 (73.1%)	8.968	**0.03**	OR = 2.413; CI: 1.35–4.32	0.030
Use of tocolytics during pregnancy	Yes 21 (20.8%) No 80 (79.2%)	Yes 10 (9.6%) No 94 (90.4%)	4.987	**0.026**	OR = 2.467; CI: 1.098–5.546	0.029
Perinatal complication (any)	Yes 67 (65.0%) No 36 (35.0%)	Yes 17 (16.5%) No 86 (83.5%)	50.254	**0.000**	9.415; CI: 4.870–18.203	0.000
Prematurity	Yes 19 (18.4%) No 84 (81.6%)	Yes 2 (1.9%) No 101 (98.1%)	15.324	**0.000**	11.42; CI: 2.586–50.455	0.001
Low birth weight (less than 2800 gr)	Yes 11 (10.7%) No 92 (89.3%)	Yes 6 (5.8%) No 97 (94.2%)	1.603	0.205		
Perinatal asphyxia	Yes 4 (3.9%) No 99 (96.1%)	Yes 1 (1.0%) No 102 (99.0%)	1.845	0.369		
Intracranial hemorrhage	Yes 6 (5.8%) No 97 (94.2%)	Yes 2 (1.90%) No 101 (98.1%)	2.081	0.279		
Neonatal jaundice	Yes 50 (48.5%) No 53 (51.5%)	Yes 10 (9.7%) No 93 (90.3%)	37.626	**0.000**	8.774; CI: 4.11–18.725	0.000
Respiratory distress syndrome	Yes 9 (8.7%) No 94 (91.3%)	Yes 2 (1.9%) No 101 (98.1%)	4.706	**0.030**	4.835; CI: 1.018–22.957	0.047
Paternal age at child’s birth	32.93 ± 6.32	31.69 ± 6.12	1.415	0.159		
Maternal age (at child’s birth)	28.45 ± 4.79	27.93 ± 5.42	0.731	0.465		

In order to explore relative effects of perinatal complications that appeared as significant predictors in univariate analyses, we performed a two-step multivariant logistic regression of ASD risk as a dependent variable. In the first step, we included prematurity, neonatal jaundice, RDS, and use of medication during pregnancy as predictors, controlling for maternal and paternal age at birth. The regression model was significant (X^2^ = 56.533, p = 0.000; Cox & Snell R^2^ = 0.256, Nagelkerke R^2^ = 0.342), with significant effects adding to the risk of ASD for all perinatal predictor variables except RDS (**Table 3**). In the second step, we explored the effects of the same predictors, adjusting not only for parental age, but for GST genotypes as well. This model was also significant (X^2^ = 62.995, p = 0.000; Cox & Snell R^2^ = 0.281, Nagelkerke R^2^ = 0.375), with prematurity and neonatal jaundice keeping their significant predictive effects, and RDS effect not showing significance again. However, after controlling for *GST* genotypes, the predictive effect of use of medication during pregnancy became insignificant (**Table 4**), leading towards the hypothesis of possible moderation by GST genotype.

**Table 4:** Two-step multivariate logistic regression model of the ASD risk with prenatal and perinatal factors as predictors.

	Step one: Controlling for parental age			Step two: Controlling for parental age and GST genotypes		
	Wald	Sig.	OR	Wald	Sig.	OR
Prematurity	4.930	**0.026**	6.093	5.043	**0.025**	6.722
Neonatal jaundice	25.548	**0.000**	8.453	24.972	**0.000**	8.814
Respiratory distress syndrome	0.549	0.459	1.926	0.236	0.627	1.545
Use of medication during pregnancy	4.175	**0.041**	2.080	3.565	0.059	2.007

A line of moderation analyses were conducted with ASD status as outcome, use of medication during pregnancy as a predictor, and each GST genotype as a moderator, controlling for other perinatal factors (neonatal jaundice, prematurity, RDS), parental age at birth, and all the other GST polymorphisms in each analysis. We found significant effect of interaction between *GSTM1* genotype and medication use during pregnancy on the risk of ASD. Therefore, GSTM1 genotype was a significant moderator of the effect of medication use during pregnancy on ASD risk. The use of medication was significantly predictive of the higher ASD risk only in carriers of *GSTM1-null* genotype, whereas among carriers of *GSTM1-active* genotype, the predictive effect of medication use was not significant (**Table 5**).

**Table 5:** Significant GSTM1 null moderation of the effects of medication use during pregnancy on ASD development (with parental age, neonatal jaundice, prematurity, RDS, and other GST genotypes as covariates; *p < 0.05; ** p < 0.01).

Predictor to outcome	Interaction effect of *GSTM1* genotype with use of medication during pregnancy B (LLCI-ULCI)	*GSTM1-active* conditional effect B (LLCI-ULCI)	*GSTM1-null* conditional effect B (LLCI-ULCI)
Use of medication during pregnancy to ASD status	−1.580 (−3.120 to −0.039)*	−1.060 (−1.1720 to 0.960)	1.474 (0.404 to 2.544)**

Moderation analyses were also conducted with ASD status as outcome, use of tocolytics during pregnancy as a predictor, and GST genotypes as moderators, with controlling for the same factors as in previous moderation analyses. A significant effect of interaction between *GSTM1* genotype and the use of tocolytics during pregnancy on the risk of ASD was also found. As for the use of all medications, the use of tocolytics was predictive of the higher ASD risk only in carriers of *GSTM1-null* genotype (**Table 6**).

**Table 6:** Significant GSTM1 null moderation of the effects of tocolytic use during pregnancy on ASD development (with parental age, neonatal jaundice, prematurity, RDS, and other GST genotypes as covariates; *p < 0.05; **p < 0.01).

Predictor to outcome	Interaction effect of *GSTM1* genotype with use of tocolytics during pregnancy B (LLCI-ULCI)	*GSTM1-active* conditional effect B (LLCI-ULCI)	*GSTM1-null* conditional effect B (LLCI-ULCI)
Use of tocolytics during pregnancy to ASD status	−2.792 (−5.208 to −0.376)*	−0.598 (−0.532 to 1.412)	2.732 (0.860 to 4.604)**

## Discussion

The present study explored the prenatal factors and perinatal complications in individuals with ASD, as well as their possible interaction with genetic polymorphisms in GSTs. Our findings have shown that prematurity, neonatal jaundice, RDS and use of medication during pregnancy were significantly more frequent in ASD group. After performing the multivariant logistic regression analysis, exploring the relative effects of individual complications, the findings showed that prematurity, neonatal jaundice, as well as the use of medication during pregnancy had a significant effect, raising the risk of ASD development. When adjusted for GST genotypes, we found no evidence of change in the effect significance for prematurity and neonatal jaundice as risk factors in ASD. However, the effect of medication use during pregnancy was moderated by GSTM1 genotype. It was significantly predictive of ASD risk only in carriers of *GSTM1-null*, whereas among carriers of *GSTM1-active* genotype no significant relationship was found between the medication use and the ASD risk. The same finding was reached when we explored the effect of tocolytics—their use was significantly predictive of ASD risk only in carriers of *GSTM1-null* genotype.

Prematurity is recognized as a significant risk factor for ASD, after controlling for other perinatal complications, as well as GST genotypes in our study. This is in line with the existing data (5, 12, 18), although our study showed even higher risk. On the other hand, a study by Burstyn et al. in which the cut-off was also set at 37^th^ week failed to confirm this association (21). As it was already mentioned, DWMI is one of the most common brain injury in prematurely born children, and is associated with oxidative stress and decreased cognitive abilities, as well as with behavioral and psychological difficulties (28, 29). *In vitro* and animal studies have shown that oxidative stress affects apoptosis and leads to decrease in myelinization and oligodendrocyte differentiation (29).

We also confirmed that neonatal jaundice is a significant risk factor for ASD, which is in concordance with several studies. Neonatal jaundice is the result of immaturity of the liver and its functions, as well as increased fetal erythrocytes degradation, while the accumulated bilirubin might potentially lead to brain damage (25). Interestingly, it has been proposed that oxidative stress might be among the primary causes of eritrocyte membrane impairment with consequent hyperbilirubinaemia. Indeed, increased superoxide dismutase (SOD) and glutathione peroxidase (GPX) activities were found in children with neonatal jaundice. A study by Raicevic et al. (2014) explored the levels of oxidative stress markers and bilirubin in children who had fetal distress during labour and it showed significantly lower eritrocyte count and significantly higher bilirubin levels. They proposed that oxidative distress might cause higher erythrocytes degradation due to fetal distress caused by other perinatal complications or primarily decreased activity of antioxidant enzymes, while increased levels of bilirubin might also act harmfuly to neonatal brain (45). Other studies have also suggested that oxidative stress is the mediator of neurotoxic effect of bilirubin (46). It has been shown that neonate carriers of *GSTM1-null* genotype are at high risk to develop pathologic hyperbilirubinemia and may have higher bilirubin levels (47). Glutathione S-transferases can act as intracellular binding proteins for nonsubstrate ligands, including bilirubin and bilirubin conjugates, thus decreasing the efflux of bilirubin into plasma. Specifically, polymorphisms in GSTM1 and GSTT1 genes may affect their ligandin functions in bilirubin transport. Our findings have shown that neonatal jaundice leads to a higher increase in risk for ASD than in recent studies. This could partially be explained by recall bias, since it seems that recalling to perinatal complications is higher in parents of children with developmental difficulties. In our group of ASD individuals, the significant effect of neonatal jaundice has not changed after controlling for genotypes.

Regarding RDS, our result showed almost five-fold increased risk of ASD, which is in agreement with literature data (17, 19). However, susceptibility for ASD development in children with RDS was somewhat lower than in our study (17, 19). A recent meta analysis also recognized RDS as a significant risk factor for ASD (48). The RDS is significantly more frequent in prematurely born children (26), but is also associated with different perinatal complications and oxidative stress. Moreover, RDS is also related to oxidants/antioxidants imbalance, since increased markers of oxidative stress were found in newborns with this condition (26, 27). In our study, after controlling for other perinatal complications, the effect of RDS became insignificant in the development of ASD.

Similarly to a previous study, we showed an increased risk of ASD development in relation to using any medication in pregnancy (49). In our study, most of the used medications were tocolytics, progesterone, antibiotics, and benzodiazepines. When we stratified the study group according to specific drugs, only tocolytics were recognized as a significant risk factor for ASD. The tocolytics used in our sample were hexoprenaline and phenoterole, beta-2 adrenergic agonists, of which the exact mechanism how they can contribute to ASD risk is not clear yet. Animal studies that explored the effect of prolonged treatment with phenoterole confirmed the increased production of free radicals; however, the studies were oriented towards cardiomyocites, and not neurons (50). *In vitro* studies, on the other hand, showed that phenoterol might be considered as a substrate for peroxidase, further producing reactive metabolytes, although its main detoxification metabolic pathway is *via* conjugation (51).

To our knowledge, our study represents the first comprehensive analysis of prenatal and perinatal complications in conjunction with oxidative stress-related gene interactions in the development of ASD. So far, this mechanistic link has been evaluated in animal models of psychiatric disorders such as schizophrenia, but not specifically in ASD (52).

A recent study conducted within a student population suggested that an adverse intrauterine and/or early life environment, accompanied by the cumulative exposure to perinatal complications, correlate with externalizing problems particularly in childhood and adolescence. This was further accompanied by increased levels of lipid peroxidation, thus pointing out to the role of oxidative distress on psychopathology in this vulnerable life period (53).

When it comes to oxidative stress-related gene interactions, our study suggests that *GSTM1* genotype moderates the effect of medication use during pregnancy on the risk of ASD, specifically for the use of tocolytics. Namely, significant predictive value on ASD risk was observed for children carriers of *GSTM1-active* genotype whose mothers used any medication during pregnancy, and specifically tocolytics, as well. This finding might be significant, since studies have shown higher risk of ASD in children whose mothers used beta-adrenergic agonists as tocolytics during pregnancy (54). To our knowledge, this finding represents the first oxidative stress-specific gene–environment interaction shown in children with ASD. This seems plausible having in mind that GSTs participate in conjugation of endogenous and exogenous xenobiotics, including medications, thus decreasing their toxicity and facilitating their excretion from the body (55). Due to deletional polymorphism, individuals that lack GSTM1 isoenzyme activity might have altered capacity for detoxification of substrate drugs, but also decreased antioxidant capacity (56, 57). In this line, it might be speculated that children carriers of *GSTM1-null* genotype are more vulnerable to drug side-effects due to impaired detoxification and/or antioxidant capacity. This further implies that different oxidative stress-related genetic and environmental factors might, in conjunction, lead to development of ASD.

It is also important to emphasize that, while the effect of medication during pregnancy is significantly moderated by *GSTM1* genotype, the effect of neonatal jaundice and prematurity increases the risk of ASD, independently of the GST genotype status. The independent effect of neonatal jaundice might be explained by the fact that different GST classes bind bilirubin with differential affinity (58). On the other hand, preterm children have decreased antioxidant defense mechanisms, due to the fact that the physiological maturation of antioxidant capacity occurs at the end of gestation (59). Therefore, preterm children might be more sensitive to neonatal oxidative stress due to immaturity of the antioxidant system, possibly regardless of their genotype.

Finally, it should be noted that the effect of perinatal complications on the ASD risk explored in our sample might be moderated by polymorphisms of other oxidative stress genes.

In our study, there were no case–control differences for LBW, perinatal asphyxia, and intracranial hemorrhage. Several studies have confirmed birth weight lower than 2,500 g to be a significant risk factor for ASD (21, 22). In our study, the criterion for LBW was 2,800 g, which is a somewhat higher cut-off, and might be an explanation for the difference. Also, the study by Haglund and Kallen showed that LBW increased the risk for autism and not for Aspergers syndrome (22). Our study included the whole autism spectrum. On the other hand, study by Mamidala et al. showed no significant correlation of LBW and ASD, which is in corcordance with our study (5). We did not find the association between risk of ASD and perinatal asphyxia. The available data are rather inconsistent, although several studies suggested the association (5, 12, 20). In our study group, perinatal asphyxia was present in 3.9% cases and 1% of controls, reaching four-fold increased ASD risk; however, this perinatal complication was not recognized as a significant risk factor. The incidence of perinatal asphyxia is 5–10 in 1,000 live born children, so it can be assumed that in a large sample its role in ASD susceptibility might reach statistical significance (60). Intracranial hemorrhage was present in 5.8% cases and 1.9% controls, reaching three-fold increased OR for developing ASD, still statistically insignificant. The literature data on this prenatal factor are also conflicting. Until now, two studies showed an increased risk of ASD, when this complication was studied individually (17) or together with cerebral oedema and convulsions (19), while Duan et al. defined the complication as intrapartal craniocerebral injury and did not find the association with ASD development (12).

There are several limitations of our study. The first is related to the relatively small sample size, since larger sample could have offered possibility to include more predictors in the multivariate analyses. Furthermore, several factors that were assessed in the study could not be explored in terms of multivariate effects due to low frequences within the groups of subjects. Also, the case–control study design does not provide possibility for causal conclusions, which could be better provided by a longitudinal design. Finally, a significant limitation is indeed the retrospective assessment of prenatal and perinatal predictor variables, which could have resulted in incorrect information due to the recall bias.

On the other hand, our study showed significant findings regarding the influence of perinatal complications on the risk of ASD, mostly confirming the findings of previous studies on this matter. Further, by controlling for GST genotypes, we also tested the hypothesis that some of the prenatal and perinatal complications have significant effect on ASD only in individuals at genetic risk, in terms of oxidative stress. A very significant result of the moderating effect of GSTM1 genotype on the effect of medication use during pregnancy offers some therapeutic possibilities to individuals at risk. Several studies explored application of antioxidant therapy in autism, such as N-acetyl cysteine, methyl B12, and omega-3 fatty acids, however with conflicting results (61–63). The overall suggestion is that not all individuals with ASD would benefit from antioxidant therapy. Indeed, only children with oxidative stress-specific susceptibility, confounding effect of prenatal and perinatal risk factors, and/or oxidative stress-related genetic polymorphisms might be the target population.

## Data Availability

The raw data supporting the conclusions of this manuscript will be made available by the authors, to any qualified researcher.

## Ethics Statement

This study was carried out in accordance with the principles of good research practice with written informed consent from all subjects or subject’s parent/caregiver. All subjects gave written informed consent in accordance with the Declaration of Helsinki. The protocol was approved by the Ethics Committee of the Institute of Mental Health, University Children’s Hospital and Faculty of Medicine, University of Belgrade, Serbia.

## Author Contributions

VM-M, MP-M, MP-E, TP, and TS designed the study. TP, MM-V, and VM-M performed the statistical analysis. VM-M, MP-M, MM-V, and MD recruited and screened the participants. MP-M diagnosed the patients. AS-R performed the genetic analyses. VM-M, MP-M, MP-E, AS-R, ME, MM-V, and RG performed the literature search and wrote the manuscript. DL-T, TP, and TS gave critical comments to the manuscript. All of the authors approved the final manuscript.

## Conflict of Interest Statement

The authors declare that the research was conducted in the absence of any commercial or financial relationships that could be construed as a potential conflict of interest.
