# Supplementary material for: Autism Spectrum Disorders and Perinatal Complications—Is Oxidative Stress the Connection?
Source: Front Psychiatry. 2019 Sep 25;10:675. doi: 10.3389/fpsyt.2019.00675 (PMC6798050; doi:10.3389/fpsyt.2019.00675)
Supplement: Supplementary file 1 [file DataSheet_1.docx]

**Sociodemographic and exposure questionnaire – mother**

**Sociodemographic and exposure questionnaire – mother**

1. Code _______________________________
2. Year of birth _______________________________
3. Age at delivery ________________________
4. Parity _____________________
5. Interpregnancy interval (except for the first pregnancy) ____________________________________________________________
6. Place of birth ________________________________
7. Place of living ______________________________
8. Level of education ______________________________
9. Current job ____________________________
10. Job before or during pregnancy _________________________
11. If you are working with chemicals, please list the chemicals that you have been in contact with ____________________________________________________________
12. For how long have you been in contact with the chemicals? ____________________________________________________________
13. Have you been in contact with those chemicals during pregnancy?

YES NO

1. If the answer is YES, during which period of pregnancy have you been in contact with those chemicals?

______________________________________________________________

1. Have you been suffering from an infectious disease during pregnancy?

YES NO

1. If the answer is YES, which disease was it? ­­­­­______________________________
2. Has the child suffered from an infectious disease immediately after delivery?

YES NO

1. If the answer is YES, which disease was it?

__________________________________________________________________

1. How old was the child at the time? ______________________________________
2. Are you a smoker? YES NO

(smoking at least 1 cigarette a day, at least during 1 year)

1. If the answer is YES, how many cigarettes a day do you smoke? ___________________
2. How long have you been smoking?__________________________________
3. If you quit smoking, for how long have you been smoking?____________________
4. How many cigarettes a day did you smoke during this period? __________
5. Did you smoke cigarettes during pregnancy? YES NO
6. If the answer is YES, how many cigarettes did you smoke a day? _______________
7. During which period of pregnancy did you smoke? _____________________________
8. If you did not smoke during pregnancy, have you been living in rooms where others smoked, during pregnancy (passive smoking) YES NO
9. Do you drink alcohol? YES NO
10. Please note how often you drink alcohol

Every day Weekly Several times a week

1. Have you been drinking alcohol during pregnancy? YES NO
2. During which period of pregnancy did you drink alcohol? ____________________
3. Did you use medication during pregnancy? YES NO
4. If the answer is YES, which medication did you take?
5. ___________________________________________
6. ___________________________________________
7. ___________________________________________
8. For how long have you been taking those medications?
9. _________________________________________
10. ___________________________________________
11. ___________________________________________
12. During which period of pregnancy were you taking those medications?
13. _________________________________________
14. ___________________________________________
15. ___________________________________________
16. Do you have any somatic illness?

_______________________________

(e.g. hypertension, diabetes)

1. Did you have this illness during pregnancy?

YES NO

**Sociodemographic and exposure questionnaire – father**

1. Code _______________________________
2. Year of birth _______________________________
3. Age at child's birth ________________________
4. Place of birth ________________________________
5. Level of education ______________________________
6. Current job/job before retirement _________________________
7. If you are wokring with the chemicals, please list the chemicals that you are in contact with, and for how long__________________________________________________
8. Place of living ___________________________
9. Are you a smoker? YES NO

(smoking at least 1 cigarette a day, at least during 1 year)

1. If the answer is YES, how many cigarettes a day do you smoke? ___________________
2. How long have you been smoking?__________________________________
3. If you quit smoking, for how long have you been smoking?____________________
4. How many cigarettes a day did you smoke during this period? __________
5. Do you drink alcohol? YES NO
6. Please note how often you drink alcohol

Every day Weekly Several times a week

1. Do you have any somatic disease? _______________________________

(e.g. hypertension, diabetes)

Perinatal complications

1. Prematurity (less than 37 gestational weeks)

YES NO

1. Low birth weight (≤2500gr)

YES NO

1. Postmaturity (over 42 gestational weeks)

YES NO

1. Perinatal asphyxia

YES NO

1. Intracranial haemorrhage

YES NO

1. Neonatal jaundice

YES NO

1. Respiratory distress syndrome

YES NO

1. Hypoglycaemia (below 1.65 mmol/L)

YES NO

1. Neonatal convulsions

YES NO

1. Systemic neonatal infections (sepsis, meningoencephalytis)

YES NO

1. Pre-eclampsia in mother

YES NO

1. Convulsions in mother during pregnancy or delivery

YES NO

1. Chronic illness of the child

YES NO
